# Factors influencing territorial occupancy and reproductive success in a Eurasian Eagle-owl (*Bubo bubo*) population

**DOI:** 10.1371/journal.pone.0175597

**Published:** 2017-04-11

**Authors:** Mario León-Ortega, María V. Jiménez-Franco, José E. Martínez, José F. Calvo

**Affiliations:** 1 Departamento de Ecología e Hidrología, Facultad de Biología, Universidad de Murcia, Murcia, Spain; 2 Bonelli’s Eagle Study and Conservation Group, Murcia, Spain; Université de Sherbrooke, CANADA

## Abstract

Modelling territorial occupancy and reproductive success is a key issue for better understanding the population dynamics of territorial species. This study aimed to investigate these ecological processes in a Eurasian Eagle-owl (*Bubo bubo*) population in south-eastern Spain during a seven-year period. A multi-season, multi-state modelling approach was followed to estimate the probabilities of occupancy and reproductive success in relation to previous state, time and habitat covariates, and accounting for imperfect detection. The best estimated models showed past breeding success in the territories to be the most important factor determining a high probability of reoccupation and reproductive success in the following year. In addition, alternative occupancy models suggested the positive influence of crops on the probability of territory occupation. By contrast, the best reproductive model revealed strong interannual variations in the rates of breeding success, which may be related to changes in the abundance of the European Rabbit, the main prey of the Eurasian Eagle-owl. Our models also estimated the probabilities of detecting the presence of owls in a given territory and the probability of detecting evidence of successful reproduction. Estimated detection probabilities were high throughout the breeding season, decreasing in time for unsuccessful breeders but increasing for successful breeders. The probability of detecting reproductive success increased with time, being close to one in the last survey. These results suggest that reproduction failure in the early stages of the breeding season is a determinant factor in the probability of detecting occupancy and reproductive success.

## Introduction

Territory occupancy and reproductive parameters of territorial species have aroused great interest in population ecology and biodiversity conservation studies, since they allow researchers and managers to explain population trends in relation to ecological and disturbance covariates [1–3]. Recent advances in occupancy models mean that both occurrence and reproductive success parameters can be included by considering multiple states for modelling seasonal [4, 5] or multi-seasonal [6] population dynamics. Occurrence or occupancy models allow a wide range of ecological and management questions to be assessed [7], and are especially useful in studies of large areas or of inconspicuous species [8, 9]. These models account for imperfect detection (i.e. failure to detect a species when in fact the species is present [3]), and provide estimates of detection probabilities in relation to site- and survey-specific covariates [10, 11].

In the case of long-lived territorial species, estimating their occurrence is a cost-effective alternative to estimating survival parameters, given the difficulty of capturing and marking individuals [12], and breeding success is a useful estimate for determining the status of populations [13]. The occupancy of a breeding site or territory is a process influenced by environmental variables, and, in this respect, prey availability and landscape characteristics are the most relevant factors for many territorial species [14–17]. Other important factors conditioning territory occupancy are natural perturbations [18], dispersal [19] and intra- and interspecific interactions [20, 21]. Some studies have found a positive relationship between territory occupancy and the previous breeding success [4, 6, 22, 23], finding that territorial fidelity may follow the “win-stay:lose-switch” rule [24, 25]. With regards to breeding success, a wide range of factors also condition this parameter, such as weather, habitat quality, intra- and interspecific competition and age [26]. Some studies suggest that breeding success is better explained by individual quality than by territory quality [22, 27, 28], because high quality individuals have greater abilities to establish themselves in nesting sites [29] and to obtain food and care for their young [30]. Moreover, individuals may cue on their own reproductive success as a way of indirectly assessing the quality of territories [22, 31].

Occupancy models may be viewed as Markov models [6], for which the probability of territory occupancy and success depends upon previous territory state [3]. In this context, multi-state models provide the appropriate analytical framework for investigating Markovian processes in population dynamics [32]. However, to date, few examples of the application of this type of model to territorial species can be found in the scientific literature [6, 33, 34, 35]. Here, we follow a hierarchical multi-season, multi-state modelling approach to determine the probability of territory occupancy and breeding success of a long-lived, cliff-nesting territorial species, the Eurasian Eagle-owl (*Bubo bubo*) in the semiarid Mediterranean environments of south-eastern Spain (Murcia). This species is an inconspicuous, nocturnal bird of prey so estimating the probabilities of detection and occurrence is of special interest for designing monitoring and conservation programs. Nevertheless, although some studies have focused on factors affecting Eurasian Eagle-owl territorial occupancy [36–38], there is a lack of long term, integrative studies on population dynamics for this species that also account for imperfect detection. Our aim was to determine factors conditioning the occupancy and reproduction of Eurasian Eagle-owls, analysing interannual variations, the influence of prior occupancy and reproductive state of the territory and the effects of several habitat and landscape covariates. To account for imperfect detection, a repeated survey monitoring program was followed (four visits to each potential breeding territory, each season), which allowed us to estimate different detectability parameters (probabilities of detecting owls and of observing the evidence of reproduction).

## Materials and methods

### Species and study area

The Eurasian Eagle-owl (*Bubo bubo*), one of the largest owl species in the world, is distributed throughout the Palaearctic region; it is sedentary and highly territorial all year round, with home ranges that vary in size, depending on habitat structure and composition, sex and the state of health of individuals [39]. It is a monogamous and long-lived species (> 15 years in the field and > 60 years in captivity [40]), adult survival probably being attributable to the acquisition of a territory and increased foraging experience [41]. In the Mediterranean region, egg-laying typically starts in mid-December and, while clutch size can vary from 1 to 5 eggs, extremes are rare [40].

**Fig 1:**
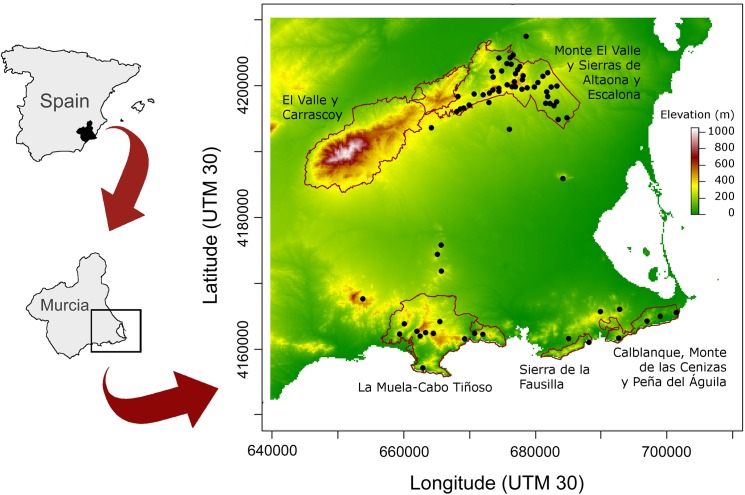
Map of the study area. Distribution of the 72 Eurasian Eagle-owl territories monitored and limits of the protected areas (Regional Parks and Special Protection Areas).

The study area is located in the east of the province of Murcia (south-eastern Spain; 37° 45’ N, 0° 57’ W; Fig 1). The climate is arid and semiarid Mediterranean with 275–400 mm of annual rainfall and an average annual temperature of 19°C. This area is a quaternary sedimentary basin surrounded by two mountainous systems. In the northern zone, a mountain chain extends from northeast to southwest, with altitudes ranging from 40 to 646 m a.s.l. It includes two protected areas: “El Valle y Carrascoy” Regional Park and “Monte El Valle y Sierras de Altaona y Escalona” Special Protection Area (SPA; site code: ES0000269). The southern limit of the basin (southern zone) is formed by a coastal massif running west to east (0–629 m a.s.l) that includes the “Calblanque, Monte de las Cenizas y Peña del Águila” Regional Park and the “Sierra de la Fausilla” (ES0000199) and “La Muela-Cabo Tiñoso” SPAs (ES0000264). The abundance of the Eurasian Eagle-owl’s main prey, the European Rabbit (*Oryctolagus cuniculus*), differs between zones [42], which is related to different land uses. In the northern zone, land is mainly dedicated to citrus and dry farming, and is home to abundant European Rabbits. In the southern zone, where the European Rabbit is much less abundant, irrigated farming is predominant, and the area includes some ancient mining sites and the important industrial area of Cartagena [43].

### Population monitoring

Between 2006 and 2012, we monitored 72 Eurasian Eagle-owl nesting territories (49 in the northern zone, and 23 in the southern zone). Previous fieldwork between 1999 and 2005 was neccesary to locate most territories and nests in the study area. Each year, we conducted four surveys to each territory during the reproductive cycle of the owls (from mid-December to mid-July). These surveys covered all the stages into which we divided the breeding period, approximately seven weeks each one: 1) courtship (December-February); 2) incubation (February-March); 3) chicks in nests (April-May); 4) fledglings (May-July). In the first stage, surveys were conducted by two teams of usually two observers, from one hour before sunset to one hour past sun down. Occupancy was determined by the usual methods used for owl censuses, including responses (e.g. elicited vocalizations, approaches) to the playback of taped calls, and the observation of signals of the species’ presence (recent faeces, pellets or prey remains) [38]. The broadcasting of conspecific vocalizations followed the methodology of Martínez and Zuberogoitia [44]. Playback sessions (territorial calls of a male) were performed at 150–250 m distance from nests, and consisted in five periods of 4 minutes each separated by periods of silence lasting 2 min [44]. The broadcast volume was adjusted to match that of live calls (~76 dB).

For the rest of the stages, surveys were conducted during daytime hours, starting approximately two hours before sunset. Evidence of reproduction (eggs or chicks) and breeding success was obtained at a distance by binoculars (x10) or telescope (x20-40) [45]. Following the terminology of Steenhof and Newton [13], a successful pair is one that raised at least one young until the minimum acceptable age for assessing success (50–60 days old in the Eurasian Eagle-owl [45]). Therefore, for each territory *i*, we elaborated a detection history vector (**h**_*i*_) of length 28 (four surveys over seven years; S1 Data), considering three occupancy states for each survey: unoccupied (state 0), occupied without successful reproduction (state 1), occupied with successful reproduction (state 2). Each territory was considered as spatially independent, and we assumed that occupancy and reproductive success of a given territory was independent from the occupancy of adjacent territories [6]. State, survey and year were used as covariates in the occupancy modelling framework (detailed below).

### Description of territory characteristics

Each potential nesting territory was characterized by five environmental covariates (Table 1). *Zone* accounted for geographical differences in landscape characteristics and global prey availability (as described in the Species and study area section); *ruggedness*, *crops* and *scrub* were used to explain physiographical and land use variability among territories; and *distance* (between nests and tracks or roads) considered the potential effects of human disturbance.

**Table 1:** Predicted relationships between human and ecological covariates and occupancy and reproduction parameters of Eurasian Eagle-owls in south-eastern Spain.

Covariate	Description	Hypothesis	Predictions for ecological parameters ^a^
State	Occupancy state of the territory the previous year: 0 (unoccupied), 1 (occupied without breeding success), 2 (occupied with breeding success).	Previous breeding success is positively correlated with a higher territory occupancy rate and reproductive success the following year.	*ψ* (++), *R* (++)
Survey	Repeated visits to each territory during the breeding period (survey 1- survey 4).	Last surveys are associated with higher occupancy and breeding success detection rates.	*ψ* (0), *R* (0) ^b^
Year	Breeding season (from 2006 to 2012).	Reproductive success is expected to vary between years.	*ψ* (0), *R* (+/-)
Zone	The study area is divided into two zones (see *Species and Study area* section): 1 (northern zone), 0 (southern zone).	The northern zone has a higher availability of prey [42], which is expected to determine a higher probability of territory occupancy and breeding success.	*ψ* (+), *R* (+)
Ruggedness	Measured as the standard deviation of altitudes (m) in a 1-km radius plot around the nest, using a 25 m digital elevation model ^c^.	Ruggedness positively influences territory occupancy [36] but negatively influences breeding success [46].	*ψ* (+), *R* (-)
Crops	Area covered by agricultural fields in a 1-km radius around nest (m^2^) ^d^.	Territory occupancy and reproductive success are negatively correlated with the presence of crops around nests.	*ψ* (-), *R* (-)
Scrub	Area covered by shrub-dominated habitats in a 1-km radius circle around nest (m^2^) ^d^.	Scrub areas favour rabbit abundance, which positively influences breeding success [47].	*ψ* (0), *R* (+)
Distance	Distance from a Eurasian Eagle-owl nest to the nearest road or forest track (m).	The proximity of roads and tracks to nests reduces the probability of territory occupancy and negatively affects breeding success.	*ψ* (+), *R* (++)

^a^ Ecological parameters are probability of occupancy (*ψ*) and probability of breeding success (*R*). The strength and direction of predicted trends are indicated with positive and negative signs. The symbol “+/-” indicates that random tendencies are expected in an ecological parameter. Zeros indicate that no relationship between occupancy or breeding success and the predictor is expected.

^b^ Survey is not related to *ψ* or *R* but is used to model detection parameters (see the *Occupancy models* section).

^c^ Data available from 1:25,000 digital elevation model map (MDT25 for Spain) (http://centrodedescargas.cnig.es/CentroDescargas/buscadorCatalogo.do?codFamilia=02107).

^d^ Data available from 1:25,000 CORINE Land Cover 2000 map (I&CLC2000) (http://centrodedescargas.cnig.es/CentroDescargas/buscadorCatalogo.do?codFamilia=02113).

Nest locations were registered by means of a GPS device and incorporated in a Geographical Information System (GRASS v.5.0.2 [48]), which allowed us to measure the environmental covariates in the nesting territories. To assess habitat characteristics, a circular plot around the nest with a radius of 1 km was established, covering an area that encompass the home range requirements of owl pairs [49]. For territories containing more than one nest, the most frequently used was considered as the territory centre. All the quantitative variables were standardized prior to analysis. For each explanatory covariate *a priori* hypotheses on their potential effects on territorial occupancy and reproductive success were established (Table 1).

### Data analysis

#### Occupancy models

Hierarchical multi-season multi-state occupancy models described by MacKenzie *et al*. [6, 33] were applied in order to estimate the ecological parameters of occupancy and breeding success in our Eurasian Eagle-owl population, that is, the probability of a territory being occupied by a breeding pair in year *t* (*ψ*_*t*_), and the probability of successful reproduction occurring in the territory given that the territory was occupied in year *t* (*R*_*t*_). Sampling sites (territories) were monitored over several seasons (7 years) during each annual breeding period. In each of these sampling seasons, the territories were surveyed a maximum of four times (see *Population monitoring* section), and assigned to one of the three occupancy states considered: unoccupied (state *m* = 0), occupied without breeding success (state *m* = 1) and occupied with breeding success (state *m* = 2). Therefore, the following ecological parameters were defined [7]: $\psi_{t}^{\left[m\right]}$, the probability of territorial occupancy in year *t*, given the previous state *m* (*m* = 0, 1 or 2 at time *t*– 1), and $R_{t}^{\left[m\right]}$, the probability of successful reproduction occurring in a territory in year *t* given that it was in state *m* in year *t*– 1 and was occupied in year *t*. For example, $\psi_{t}^{\left[0\right]}$ is the probability of a territory being occupied in year *t* given that it was unoccupied in year *t*– 1, and $R_{t}^{\left[2\right]}$ is the probability of successful reproduction in year *t*, given that it was occupied and had successful breeding in year *t*– 1.

We also modelled the probabilities of detection of occupancy and breeding success following the parameterization of Nichols *et al*. [4], where $p_{t,j}^{\left[1\right]}$ is the probability of detecting occupancy in the survey *j* of year *t*, given that the territory was occupied without successful breeding in year *t*; $p_{t,j}^{\left[2\right]}$ is the probability of detecting occupancy in the survey *j* of year *t*, given that the territory was occupied with successful reproduction in year *t*; *δ*_*t*,*j*_ is the probability of observing the evidence of successful reproduction in survey *j*, given that successful reproduction occurred in year *t*. The detection probabilities $p_{t,j}^{\left[1\right]}$ and $p_{t,j}^{\left[2\right]}$ were modelled based on the *survey* covariate (courtship, incubation, chicks in nests, fledglings) but, for simplicity, they were considered constant during the study period (with no variation between years: $p_{1\text{-}4}^{\left[1\right]}$ and $p_{1\text{-}4}^{\left[2\right]}$), since preliminary models indicated that this was the best option. Evidence of successful reproduction (*δ*_*t*,*j*_) can only be assessed from the second survey onwards (according to Eurasian Eagle-owl breeding phenology), so it was modelled as zero for the first survey (*δ*_1_ = 0) and allowed to vary independently for the rest of the surveys in a given season but considered constant across years (*δ*_2–4_).

The probabilities $\psi_{t}^{\left[m\right]}$ and $R_{t}^{\left[m\right]}$ were modelled as linear-logistic functions of the covariates listed in Table 1. For example:

$$\text{logit}\;\left(\psi_{i,\;t+1}^{\left[m\right]}\right)=\beta_{0}+\beta_{\text{state}}\times {\mathrm{state}}_{m}+\beta_{\mathrm{distance}}\times {\mathrm{distance}}_{i}$$ (1)

where *β*_0_ is the intercept with the average value for all territories, *β*_*state*_ is the parameter that accounts for the effect of the previous state *m* (*state*_*m*_ *=* 0, 1 or 2), and *β*_*distance*_ is the slope parameter for the relationship with distance from a given nest (site *i*) to the nearest road or forest track (*distance*_*i*_).

#### Model selection

Model selection was based on the Akaike Information Criterion (AIC) [50, 51]. In order to identify clearly how the covariates of ecological parameters influence the AIC of each model, $\psi_{t}^{\left[m\right]}$ and $R_{t}^{\left[m\right]}$ were modelled according to different combinations of the hypotheses of Table 1. Given the very large number of models that would have to be fitted to the data (representing different combinations of hypotheses about occupancy and breeding success), we followed the two-phase approach outlined by MacKenzie *et al*. [32]. First, the influence of the covariates on one parameter (for example $\psi_{t}^{\left[m\right]}$) was modelled until the best model was obtained. Second, the best model obtained for that parameter was fixed in order to model the influence of the covariates on the other parameter (for example $R_{t}^{\left[m\right]}$). The ΔAIC for the *i*th model was computed as AIC_*i*_ − min(AIC). Models with ΔAIC < 2.0 were considered alternative models to the selected model, but examined to detect potential uninformative parameters [51]. We also used the weight of AIC (*w*) as a measure of relative importance for each model, so that all the weights for all models added up to 1 [50]. The analyses were performed with the software PRESENCE 10.6 [52]. Parameter estimates are provided with their unconditional standard errors [50].

### Ethics statement and permits

Authorization for the study was provided by the Dirección General de Medio Ambiente of the Autonomous Community of Murcia, which regulates the management of wildlife and endangered populations in the study area. Most of the owl territories monitored in the study were located on public land. In the case of the territories located on private land, access was granted by landowners. This study forms part of a wider investigation on the ecology of Mediterranean populations of the Eurasian Eagle-owl [42, 43, 53].

## Results

### Occupancy probabilities

**Fig 2:**
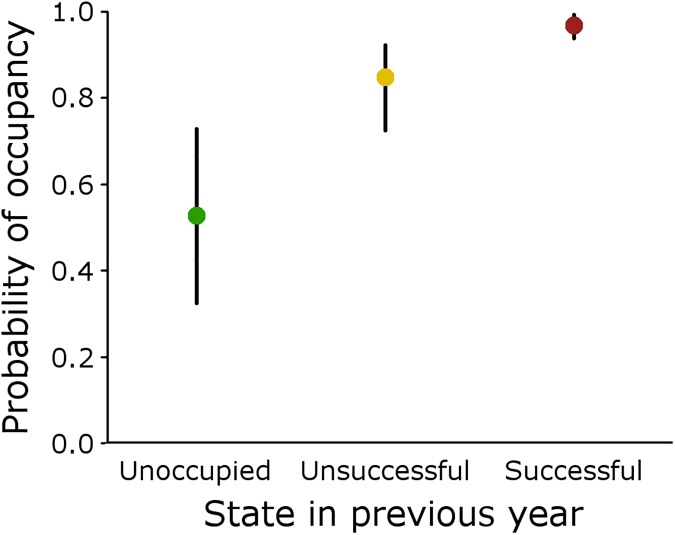
Probabilities of territorial occupancy by Eurasian Eagle-owls given the occupancy state of a territory in the previous year. Model-averaged estimates of the probability of territory occupancy ($\psi_{t}^{\left[m\right]}$) from models considering the occupancy state of the territory in the previous year: unoccupied (state *m* = 0), occupied without breeding success (state *m* = 1) and occupied with breeding success (state *m* = 2).

An analysis of the territorial occupancy dynamics showed that the probability of a territory being occupied in a given year is mainly determined by its occupancy status in the previous year (S1 Table). State was the only covariate included in the best model (*w* = 0.48), and it was also included in the two alternative models (ΔAIC < 2.0; Table 2). The estimated model-averaged occupancy probabilities were 0.53 (95% CI: 0.32, 0.73) for territories unoccupied in the previous year, 0.85 (95% CI: 0.72, 0.92) for occupied territories but without successful reproduction, and 0.98 (95% CI: 0.94, 0.99) for territories with successful reproduction (Fig 2).

**Table 2:** Top-ranked models from the analysis of Eurasian Eagle-owl territorial occupancy ($\psi_{t}^{\left[m\right]}$).

Model	*K*	AIC	ΔAIC	*w*	Deviance
*ψ*(State)	25	2193.86	0.00	0.4778	2143.86
*ψ*(State + Crop)	26	2194.55	0.69	0.3384	2142.55
*ψ*(State + Ruggedness)	26	2195.85	1.99	0.1767	2143.85

Summary of the model selection procedure on dynamic occupancy probabilities. Only models with ∆AIC < 2.0 are reported. *K* is the number of parameters in the model, AIC is the Akaike Information Criterion, ΔAIC is the relative difference in the AIC values, and *w* is the Akaike weight. Following the two-phase approach outlined in the methods section, the probabilities of breeding success, *R*, were modelled considering the influence of annual variation, the previous state of the territory and the ruggedness of the territory *R*(Year + State + Ruggedness). The probabilities of detecting occupancy given that the territory was occupied without successful breeding ($p_{1\text{-}4}^{\left[1\right]}$) and detecting occupancy given that the territory was occupied with successful reproduction ($p_{1\text{-}4}^{\left[2\right]}$) were modelled based on the *survey* covariate but considered constant across years. The probability of detecting a successful reproduction was fixed as zero for the first survey (*δ*_1_ = 0) and allowed to vary independently for the rest of the surveys, but considered constant across years (*δ*_2–4_).

The two alternative models suggest an additional influence of two environmental covariates on the probability of territory occupancy: *crops* and *ruggedness*. However, in the second case, model deviance is not reduced (Table 1), which indicates that this is not a competitive model [51]. The model including crops has an Akaike weight close to that of the best model, and indicates a positive relationship between the area covered by crops in the territory and the occupancy probability (*β*_*crops*_ = 0.26, SE = 0.23). Our models showed no evidence of annual variations in the probabilities of territorial occupancy, nor of differences between the northern and southern zones of the study area (S1 Table).

### Reproduction probabilities

**Fig 3:**
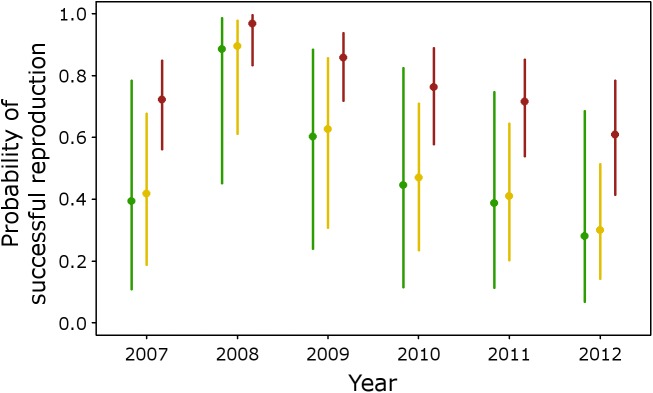
Probabilities of successful reproduction by Eurasian Eagle-owls given the occupancy state of a territory in the previous year. Estimated annual probabilities of breeding success ($R_{t}^{\left[m\right]}$) from the best reproductive model, considering territories unoccupied in the previous year (state *m* = 0; green dots), occupied without breeding success in the previous year (state *m* = 1; yellow dots) and occupied with breeding success in the previous year (state *m* = 2; red dots). Vertical lines represent unconditional 95% confidence intervals.

**Fig 4:**
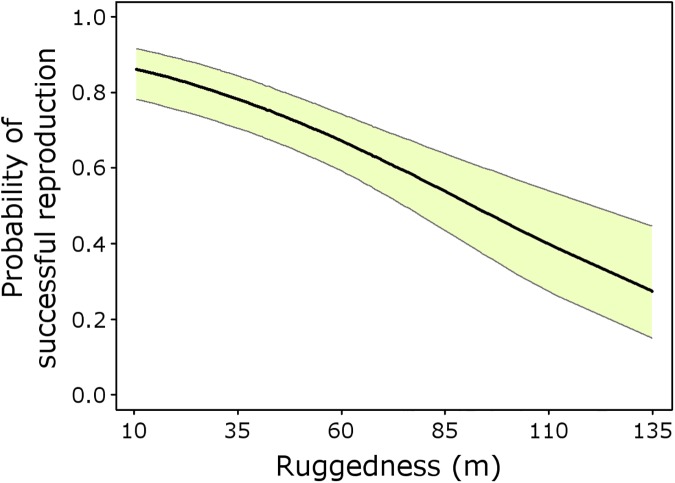
Probability of successful reproduction by Eurasian Eagle-owls related with territory ruggedness. Estimated probabilities of breeding success (*R*) from the best reproductive model depending on territory ruggedness (measured as the standard deviation of altitudes in a 1-km radius plot around the nest). Light green shading represents the unconditional 95% confidence interval of the regression line.

The best explanatory model from the analysis of breeding success in Eurasian Eagle-owl (S2 Table) was *R*(Year + State + Ruggedness), which indicates annual variations in reproductive success and dependence of the previous state of the territory (Fig 3), and a negative influence of ruggedness (*β*_*ruggedness*_ = -0.64, SE = 0.14; Fig 4). This best model has strong support (*w* = 0.84) and, based on AIC differences and Akaike weights, no alternative models merit consideration (S2 Table).

### Detection probabilities

In general, the detection parameters $p_{1\text{-}4}^{\left[1\right]}$ and $p_{1\text{-}4}^{\left[2\right]}$ estimated for the four surveys to model the ecological parameters $\psi_{t}^{\left[m\right]}$ and $R_{t}^{\left[m\right]}$ were high (Table 3). Detectability decreased with time for unsuccessful breeders but increased in the case of successful breeders. These detection probabilities determined that the overall average estimated probability of territory occupancy (all states and years) was 0.1 higher (0.97; 95% CI: 0.93, 0.99) than the naïve estimate (0.87; 95% CI: 0.85, 0.90).

**Table 3:** Estimates of detection probabilities for Eurasian Eagle-owls.

	Survey			
Detection parameter	1	2	3	4
$p_{1\text{-}4}^{\left[1\right]}$	0.75 (0.62–0.84)	0.64 (0.53–0.74)	0.50 (0.39–0.62)	0.28 (0.18–0.42)
$p_{1\text{-}4}^{\left[2\right]}$	0.70 (0.63–0.77)	0.89 (0.84–0.92)	0.95 (0.91–0.97)	0.96 (0.90–0.98)
*δ*_1_	0.00	–	–	–
*δ*_2–4_	–	0.02 (0.01–0.05)	0.29(0.23–0.35)	0.93 (0.85–0.97)

Estimated detection probabilities from the best occupancy and reproductive model, considering only influence of the *survey* covariate. The parameters $p_{j}^{\left[1\right]}$ and $p_{j}^{\left[2\right]}$ are the probabilities of detecting the species in survey *j* given that the territory was occupied without successful breeding in the previous year, or with successful breeding in the previous year, respectively. The parameter *δ*_*j*_ is the probability of observing evidence of successful reproduction in survey *j*. *δ*_1_ was set to zero because successful reproduction can be only assessed from the second survey onwards. Unconditional 95% confidence intervals are shown in parentheses.

The values of the detection parameter *δ*_2–4_ showed that the probability of detecting evidence of successful reproduction increases with each subsequent survey during the breeding period in a given year, with a large difference between the third and the fourth surveys (Table 3). The value of *δ*_4_, close to 1.0, determines that the average estimated probability of reproductive success (all states and years) was not much higher (0.73; 95% CI: 0.64, 0.81) than the naïve estimate (0.67; 95% CI: 0.54, 0.79).

## Discussion

Modelling territory occupancy and reproduction dynamics is essential for understanding population processes in territorial species [6, 32]. The results obtained using multi-season, multi-state models showed high rates of territory occupation and breeding success in the Eurasian Eagle-owl population studied. The best occupancy model indicated that the factor that best explained occupancy of a given territory is its previous occupancy and reproductive status. In particular, breeding success determines high territorial reoccupation rates. The same observation has been made for other long-lived, resident or migratory birds of prey, such as the Spotted Owl (*Strix occidentalis*) [33], the Booted Eagle (*Hieraetus pennatus*) [23] or the Peregrine Falcon (*Falco peregrinus*) [35]. Territorial fidelity is one of the most influential factors conditioning the dynamics of territorial occupation [22, 25,54], and is apparently determined by the experience of the individual birds [55]. Reproductive experience has been related to the individual’s age in long-lived species [56], and experienced individuals tend to occupy high quality territories [57]. In contrast, younger individuals tend to be more inexperienced and occupy territories of poorer quality, which often do not favour continued occupation so they may be forced to seek a suitable territory in other areas [58]. Although our study does not present direct evidence for this type of relationship, previous studies with radio-tagged individuals in our study area [53] suggest that the Eurasian Eagle-owls frequently use the same territories in successive years with few territory changes.

Some studies have found that the landscape features and habitat characteristics are good predictors of territory occupancy in Eurasian Eagle-owl populations [36, 47, 58]. Our results also suggest the influence of habitat covariates on the probabilities of territory occupation. More specifically, the best models showed a positive relationship between the area covered by crops in the territory and the probability of occupancy. By contrast, our models showed no evidence of annual variations in the probabilities of territorial occupancy, or of differences between the northern and southern zones of the study area, which suggests that spatial and temporal changes in the abundance of rabbit, the main prey species of Eurasian Eagle-owls, has little influence.

Moreover, although a negative effect of human activities in the proximity of nests may be expected for many raptor and owl species [59–61], we found no evidence of a relationship between territory occupation and human disturbance in our study area. Compared with other large birds of prey, Eurasian Eagle-owls, with their cryptic and nocturnal behaviour, seem to show an acceptable tolerance of human proximity and human-induced environmental disturbance [45, 46].

The best model of reproductive success for Eurasian Eagle-owls included year, previous territorial state and ruggedness as explanatory covariates. Long-term monitoring programs of raptor and owl species show that annual variations in breeding success are more important than variations in territorial occupancy rates [23, 33, 62], which are mainly attributed to fluctuations in the availability of prey species [63–65]. In Mediterranean habitats, climatic conditions and disease may cause important interannual density variations in the main prey of the Eurasian Eagle-owl, the European Rabbit [66]. Such annual variations are therefore an important factor determining the number of owl breeding pairs and productivity [67–69], as well as annual population growth rates [70]. Nevertheless, the probability of reproductive success in a given territory is also strongly conditioned by its occupancy and reproductive state in the previous year. As discussed above, experienced individuals tend to occupy high quality territories, and this circumstance is expected to increase the probability of reoccupation and breeding success. This is clear from Fig 3, which shows large differences in reproductive success probabilities between territories occupied with breeding success in the previous year and unoccupied territories or occupied without breeding success. This dependency of both occupancy and reproductive success on previous territory state suggests that the system follows some type of Markov process [6, 21, 71].

The other factor influencing reproductive success in the population studied is the ruggedness of territories. However, while our best occupancy models showed a slight positive relationship with territory ruggedness, the best reproductive model showed a stronger, but negative relationship with this covariate. Previous studies with the Eurasian Eagle-owl, carried out considering different spatial scales, have also provided this kind of contradictory result. Some studies have shown that topographic irregularity is a factor determining the presence of owl nests, and that the species has a preference for steep terrains as safe breeding places [36, 38, 47]. However, other studies pointed to a preference for small cliffs for breeding, with easy access to high densities of profitable prey such as rabbits and rats [45, 49, 58]. Eurasian Eagle-owls have small home ranges and exhibit a movement pattern based on short flights [39], so the location of nests on flat lands and close to foraging areas, could facilitate the frequent ascent flights and transfer of prey to the nest by breeding individuals, and therefore the optimization of energy costs derived from foraging activity [46].

Interestingly, our analyses found no evidence of human influence on reproductive success, which contrasts with the large number of studies showing a negative relationship between human disturbance and breeding success in many raptor and owl populations. For example, Margalida *et al*. [72] found that the probability of nest abandonment by Cinereous Vultures (*Aegypius monachus*) was dependent on the distance of workers from the nest and the level of noise of their activities. Even for human-tolerant species, such as the American Kestrel (*Falco sparvarius*), much-travelled roads negatively affect its reproductive success [73]. The lack of relationship found in our study could be explained by the low degree of interaction of Eurasian Eagle-owls with humans as a consequence of their nocturnal habits and the difficulty of detecting the species given its inconspicuous behaviour, which may also hinder monitoring of the population. In this respect, accounting for imperfect detection is a necessary task for modelling population processes, even in highly intensive studies [74]. However, the results obtained in our study showed that the estimated parameters of occupancy and reproductive success were not much higher than the naïve (observed) estimates. Our models indicate that occupancy detection probabilities vary depending the reproductive status of the territorial pair, which is high and increasing from courtship to incubation for successful pairs, but notably decreases in the last surveys when reproductive failure has occurred. During the courtship period the species exhibits a strong territorial behaviour through vocalizations [44, 75] and marking signposts with faeces and the remains of prey to communicate with conspecifics [76]. This signalling behaviour is not usually maintained in pairs that fail to reproduce, so they tend to go more unnoticed than successful pairs [76]. On the other hand, estimates of the probabilities of detecting reproductive success (which we fixed at 0 for the first survey) increase substantially from the second to the last visit, reaching a value close to one.

Summarizing, for our Eurasian Eagle-owl population, the probabilities of territory occupancy and reproductive success are mainly determined by the territorial status of the previous year, but with strong annual variations in reproductive success, and a minor influence of some habitat features. The Markovian nature of the occupation and reproduction processes points to the importance of individuals’ quality in the selection of a territory, given that owls may use their own reproductive success to assess the quality of territories [29, 31, 77]. Hence, past success and individual experience would have a greater influence on territorial occupancy dynamics of the Eurasian Eagle-owl than other variations resulting from factors related to climate, habitat and human activity.

## Supporting information

S1 Data Input data for PRESENCE. Occupancy histories of 72 Eurasian Eagle-owl territories (four surveys over seven years) in south-eastern Spain, and associated environmental covariates.
(TXT)

S1 TableSummary of model selection process on dynamic occupancy probabilities ($\psi_{t}^{\left[m\right]}$) of a Eurasian Eagle-owl population in south-eastern Spain.(PDF)

S2 TableSummary of model selection process on dynamic reproduction probabilities ($R_{t}^{\left[m\right]}$) of a Eurasian Eagle-owl population in south-eastern Spain.(PDF)
