# Supplementary material for: Factors influencing territorial occupancy and reproductive success in a Eurasian Eagle-owl (Bubo bubo) population
Source: PLoS One. 2017 Apr 11;12(4):e0175597. doi: 10.1371/journal.pone.0175597 (PMC5388503; doi:10.1371/journal.pone.0175597)
Supplement: S1 Table — (PDF) [file pone.0175597.s002.pdf]

S1 Table. Summary of model selection process on dynamic occupancy probabilities ($\psi_t^{[m]}$) of a Eurasian Eagle-owl population in south-eastern Spain.

**Model**	***K***	**AIC**	**ΔAIC**	***w***
ψ(State)	25	2193.86	0.00	0.4778
ψ(State + Crops)	26	2194.55	0.69	0.3384
ψ(State + Ruggedness)	26	2195.85	1.99	0.1767
ψ(Year + State + Ruggedness)	31	2203.28	9.42	0.0043
ψ(State + Zone)	26	2205.07	11.21	0.0018
ψ(Year + State)	30	2207.22	13.36	0.0006
ψ(Year + State + Crops)	31	2207.22	15.26	0.0002
ψ(Year × State)	40	2209.80	15.94	0.0002
ψ(Year)	28	2227.62	33.76	0.0000
ψ(.)	23	2227.81	33.95	0.0000
ψ(Year + Ruggedness)	29	2229.27	35.41	0.0000
ψ(Year + Crops)	29	2231.09	37.23	0.0000
ψ(Year + Ruggedness + Crops)	30	2232.74	38.88	0.0000
ψ(Year + State + Zone)	31	2235.49	41.63	0.0000
ψ(Zone + Crops)	24	2236.54	42.68	0.0000
ψ(Crops)	23	2236.61	42.75	0.0000
ψ(Zone)	23	2236.61	42.75	0.0000
ψ(Ruggedness)	23	2236.61	42.75	0.0000
ψ(Ruggedness + Crops)	24	2238.61	44.75	0.0000
ψ(Zone + Ruggedness)	24	2238.61	44.75	0.0000
ψ(Year + Zone + Ruggedness)	30	2241.46	47.60	0.0000
ψ(Year + State + Crops)	30	2242.60	48.74	0.0000
ψ(Year + Zone)	29	2244.30	50.44	0.0000

Summary of 23 multi-season, multi-state models for occupancy probabilities, including the total number of estimable parameters (*K*), the value of the Akaike Information Criterion (AIC), the relative differences in AIC (ΔAIC) and the Akaike weights (*w*). Models are ordered in terms of ΔAIC. *ψ*(.) denotes the null (only constant) model. The probabilities of breeding success, *R*, were modelled considering the influence of annual

variation, the previous reproductive state of the territory and the ruggedness of the territory R(Year + State + Ruggedness). The probabilities of detecting occupancy given that the territory was occupied without successful breeding ($p_{1\text{-}4}^{[1]}$) and detecting occupancy given that the territory was occupied with successful reproduction ($p_{1\text{-}4}^{[2]}$) were modelled based on the *survey* covariate but considered constant across years. The probability of detecting a successful reproduction was fixed as zero for the first survey ($\delta_1 = 0$) and allowed to vary independently for the rest of the surveys, but considered constant across years ($\delta_{2\text{-}4}$).
