# Supplementary material for: Factors influencing territorial occupancy and reproductive success in a Eurasian Eagle-owl (Bubo bubo) population
Source: PLoS One. 2017 Apr 11;12(4):e0175597. doi: 10.1371/journal.pone.0175597 (PMC5388503; doi:10.1371/journal.pone.0175597)
Supplement: S2 Table — (PDF) [file pone.0175597.s003.pdf]

S2 Table. Summary of model selection process on dynamic reproduction probabilities ($R_t^{[m]}$) of a Eurasian Eagle-owl population in south-eastern Spain.

Model	*K*	AIC	ΔAIC	*w*
R(Year + State + Ruggedness)	25	2193.86	0.00	0.8417
R(Year + Ruggedness + Crops)	24	2199.54	5.68	0.0492
R(Year + Ruggedness)	23	2199.69	5.83	0.0456
R(Year + Ruggedness + Scrub)	24	2200.75	6.89	0.0269
R(Year + Zone + Ruggedness)	24	2201.65	7.79	0.0171
R(Year + Ruggedness + Distance)	24	2201.69	7.83	0.0168
R(Year + State + Scrub)	25	2206.53	12.67	0.0015
R(Year + State + Crops)	25	2208.17	14.31	0.0007
R(State + Ruggedness)	20	2210.09	16.23	0.0003
R(Year + State + Distance)	25	2211.13	17.27	0.0001
R(Year + State + Zone)	25	2211.60	17.74	0.0001
R(Year + State)	24	2212.49	18.63	0.0001
R(Ruggedness + Crops)	18	2218.92	25.06	0.0000
R(Year + Crops + Scrub)	24	2219.33	25.47	0.0000
R(Year + Crops)	23	2219.76	25.90	0.0000
R(State + Scrub)	20	2220.18	26.32	0.0000
R(Year + Scrub)	23	2220.54	26.68	0.0000
R(Year + Distance + Crops)	24	2221.21	27.35	0.0000
R(Year + Zone + Scrub)	24	2221.28	27.42	0.0000
R(State + Crops)	20	2221.44	27.58	0.0000
R(Ruggedness + Distance)	18	2221.63	27.77	0.0000
R(Year + Zone + Crops)	24	2221.73	27.87	0.0000
R(Year + Distance + Scrub)	24	2222.27	28.41	0.0000
R(State + Distance)	20	2222.56	28.70	0.0000
R(Year × State)	34	2223.28	29.42	0.0000
R(State)	19	2223.82	29.96	0.0000
R(Ruggedness)	17	2223.87	30.01	0.0000
R(Zone + Ruggedness)	18	2225.23	31.37	0.0000
R(Year + Distance)	23	2225.24	31.38	0.0000

R(Ruggedness + Scrub)	18	2225.37	31.51	0.0000
R(Year + Zone + Distance)	24	2226.79	32.93	0.0000
R(Year + Zone)	23	2226.82	32.96	0.0000
R(Year)	22	2227.53	33.67	0.0000
R(State + Zone)	20	2229.43	35.57	0.0000
R(Zone + Scrub)	18	2239.33	45.47	0.0000
R(Distance + Crops)	18	2239.81	45.95	0.0000
R(Distance + Scrub)	18	2240.22	46.36	0.0000
R(Scrub)	17	2241.39	47.53	0.0000
R(Crops)	17	2242.25	48.39	0.0000
R(Distance)	17	2242.26	48.40	0.0000
R(Zone + Distance)	18	2242.79	48.93	0.0000
R(Zone + Crops)	18	2243.82	49.96	0.0000
R(Zone)	17	2245.04	51.18	0.0000
R(.)	17	2247.45	53.59	0.0000

Summary of 44 multi-season, multi-state models for successful reproduction probabilities, including the total number of estimable parameters (*K*), the value of the Akaike Information Criterion (AIC), the relative differences in AIC (ΔAIC) and the Akaike weights (*w*). Models are ordered in terms of ΔAIC. *R*(.) denotes the null (only constant) model. The probabilities of territorial occupancy ψ were modelled considering only the previous state of the territory ψ(State). The probabilities of detecting occupancy given that the territory was occupied without successful breeding ($p_{1\text{-}4}^{[1]}$) and detecting occupancy given that the territory was occupied with successful reproduction ($p_{1\text{-}4}^{[2]}$) were modelled based on the *survey* covariate but considered constant across years. The probability of detecting a successful reproduction was fixed as zero for the first survey ($\delta_1 = 0$) and allowed to vary independently for the rest of the surveys, but considered constant across years ($\delta_{2\text{-}4}$).
